# Postmastectomy Late Breast Reconstruction with Transverse Rectus Abdominis Flap after Primary Closure with Latissimus Dorsi

**DOI:** 10.1055/s-0044-1791765

**Published:** 2024-10-24

**Authors:** Edmilson Micali, André Mattar, Ana Claudia Benjamim Burattini, Luiz Henrique Gebrim, René Aloisio da Costa Vieira

**Affiliations:** 1 Centro de Referência da Saúde da Mulher, Hospital da Mulher, SP, Brasil; 2 Breast Surgery Department, Oncoclínicas, SP, Brasil; 3 Breast Surgery Department, Hospital Beneficiência Portuguesa, SP, Brasil; 4 Breast Surgery Department, Hospital de Câncer de Muriaé, Muriaé, MG, Brasil

**Keywords:** breast cancer, surgical flaps, locally advanced tumors

## Abstract

Locally advanced breast cancer (LABC) is common in countries where organized screening is not effective. Although neoadjuvant therapy increases resectability, many patients undergo mastectomy and, in some cases, flaps are necessary for primary closure of the chest wall. Despite a worse prognosis, some of these women will achieve long-term survival and may require breast reconstruction. The literature on the subject is scarce. We present the cases of two patients with LABC undergoing neoadjuvant chemotherapy, mastectomy with extensive soft-tissue resections in the anterior chest wall, and closure with extended V-Y latissimus dorsi (LD) myocutaneous flaps. After 2 years of follow-up, they were without recurrence. They were submitted to a delayed breast reconstruction using a bipedicled transverse rectus abdominis myocutaneous (TRAM) flap. To our knowledge, this is the first publication reporting secondary reconstruction with TRAM flap after primary closure of the chest wall with LD for LABC.

## Introduction

Nonmetastatic locally advanced breast cancer (LABC) requires a multidisciplinary approach, and neoadjuvant chemotherapy (NC) may improve local conditions for surgery. Many patients undergo mastectomy for LABC; many are submitted to primary closure, but some require thoracoabdominal flaps (TAF), and others myocutaneous flaps (MF). 1 2 3 4

The literature is scarce on delayed breast reconstructions in patients who have undergone a mastectomy and require primary skin closure using flaps. 5 When evaluating secondary reconstruction after TAF, one study reported reconstruction with a prosthesis, and another described with latissimus dorsi (LD) MF. 5 There is no case reported in the literature with patient primary submitted to LD-MF who underwent late breast reconstruction with transverse rectus abdominis myocutaneous (TRAM) flap.

## Case Reports

### Case 1

**Fig. 1:**
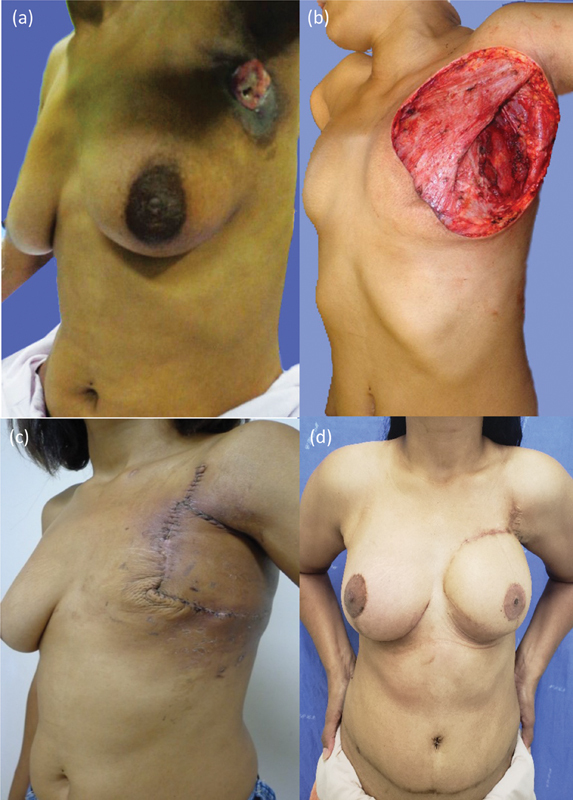
Case 1: ( **a** ) tumor, ( **b** ) resected area, ( **c** ) surgical outcome of latissimus dorsi V-Y closure, and ( **d** ) defect reconstruction with a rectus abdominis myocutaneous flap with symmetrical breast after 8.5 years.

A 32-year-old patient presented with invasive ductal carcinoma, luminal B Her-negative molecular subtype, CS IIIB, T4bN2M0, with an 11 × 9 cm ulcerated tumor in the axillary area, and extensive skin infiltration ( Fig. 1a ). She underwent NC with six cycles of the TAC regimen (docetaxel [Taxotere], doxorubicin [Adriamycin], and cyclophosphamide), showing a partial response.

Thereafter, she underwent a modified radical mastectomy ( Fig. 1b ), leaving an 18 × 18 cm wall defect, which was closed with an extended V-Y LD-MF ( Fig. 1c ). Pathological examination revealed a partial response, yT4 (7.5 cm) N1 (2/26) M0. The patient underwent adjuvant radiotherapy and hormone therapy.

After 36 months, she showed no recurrence ( Fig. 1c ), undergoing bipedicled TRAM flap, with excision of surgical closure scars up to the middle of the **V** -shaped island. This scar resection required subcutaneous tissue detachment from the chest wall, medially to the sternal edge, upward to the second intercostal space (ICS), downward to the height of the contralateral mammary fold, and laterally to the mid-axillary line. The TRAM flap was elevated with the area of anterior aponeurosis of the rectus abdominis around its periumbilical epigastric perforators to keep them intact, with approximately 8 cm in vertical length and 4 cm in width. A polypropylene mesh was used to correct the failure of bilateral aponeurosis in the donor area of the TRAM flap, and no surgical complications occurred. The patient was recurrence free 8.5 years after the primary treatment, subsequently undergoing breast symmetrization and nipple reconstruction with a CV flap 6 ( Fig. 1d ).

### Case 2

**Fig. 2:**
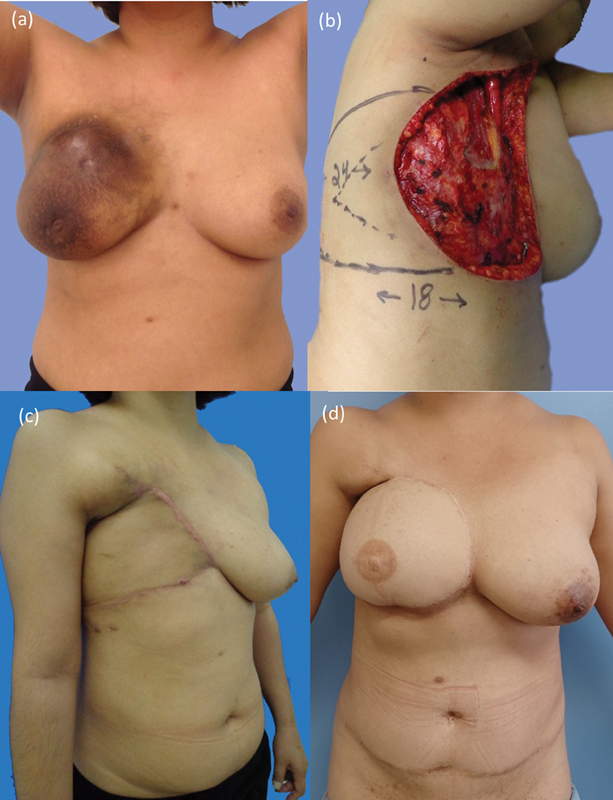
Case 2: ( **a** ) tumor, ( **b** ) area of resection, ( **c** ) primary reconstruction with a latissimus dorsi V-Y flap, and ( **d** ) late reconstruction with transverse rectus abdominis myocutaneous flap 8 years after the primary surgery.

A 31-year-old patient with invasive ductal carcinoma, triple negative, CS IIIB, T4bN2M0 (tumor area: 21 × 18 cm) underwent NC with the TAC regimen. She had a partial response and exhibited continued cutaneous involvement (15 × 14 cm) and an 8-cm residual tumor. She underwent a modified radical mastectomy and partial resection of the deep portion of the pectoralis major muscle (lesion area: 20 × 16 cm). This lesion was primarily closed using a V-Y LD-MF ( Fig. 2a–c ). Pathology revealed the lesion as yT4 (8.7 cm) N0 (0/35) M0. She underwent adjuvant chest wall and ipsilateral supraclavicular fossa radiotherapy.

No recurrence was evident 2 years postoperatively. The patient underwent bipedicled TRAM flap breast reconstruction after scar excision and skin detachments. A polypropylene mesh was used, and no surgical complications occurred. One year after breast reconstruction, she underwent nipple reconstruction with a CV flap and areola pigmentation. After 8 years of follow-up, the patient still has no recurrence and refused mammoplasty to symmetrize the left breast ( Fig. 2d ).

## Discussion

LABC is a frequent condition in developing countries, a fact related to limitations associated with diagnosis, navigation, and treatment. Oncological treatments with chemotherapy and hormone therapy demonstrate improved long-term clinical outcomes and reduced breast cancer mortality. 7 The favorable pathological response depends on tumor characteristics and NC regimen, increasing resectability and breast conserving surgery rates. However, disease progression is generally expected in these cases of LABC and, therefore, extensive mastectomies would be considered, and in some cases, flaps will be required for primary skin closure.

The lesion size influences the flap choice and in general mastectomy is associated with primary suture. Broader lesions need flaps, like TAF or MF, like LD-MF. 1 2 3 4 Higher lesions reaching to or above the clavicle are safely closed using LD-MF, external oblique MF (EO-MF), vertical rectus abdominis myocutaneous (VRAM), bilobed, or TRAM flap. 1 3 8 For axillary lesions, we can use LD-MF, VRAM, or TRAM flap.

LD-MF closes large defects, enabling rapid recovery before subsequent adjuvant treatment. 2 Moreover, the extended V-Y technique is an easy-to-execute option. 1 It is also associated with lower postoperative complication rates compared with those associated with TAF or rectus abdominis and EO-MF, making it the flap of choice for these large lesions. 2 5 The surgeon opted for LD-MF, 1 which is associated with a low rate of skin necrosis, 2 in addition to being resistant to radiotherapy.

Patients with LABC who require flap reconstruction have a significant local recurrence rate 2 ; thus, at our facility, we wait at least 1 year following radiotherapy before offering secondary reconstruction alternatives.

Studies on breast reconstruction 5 in this patient subgroup are limited because of their reduced survival. The V-Y LD-MF's ease of planning, dissection, and preparation makes it a key tool in closing the chest wall. 3 Following the first use of the LD muscle, subsequent breast reconstruction can be performed using pedicled TRAM or free flaps, like deep inferior epigastric artery perforator (DIEP). However, in our public institution we do not have material and/or human resources to perform microsurgical flaps.

Rectoabdominal flaps can be monopedicled or bipedicled, autonomized or not, horizontal, or vertical. Bipedicled flaps were chosen to provide additional perfusion, increasing the skin and fat volumes in both patients, which would not have sufficient volume for adequate reconstruction if we had opted for the monopedicled TRAM flaps. 9 10 Although both rectus abdominis muscles have a significant impact on the abdominal dynamics, and can lead to a hernia or a bulge, a polypropylene mesh was used for donor site repair, and no late complications were observed.

## Conclusion

Improvements in the prognoses of breast cancers, including LABC, necessitate surgical approaches that preserve all abdominal wall structures. LD and MF are effective, allowing primary closure alternatives for significant chest wall lesions, owing to their good coverage of large defects, low necrosis rates, and ease of performance. They also allow, in selected cases, a second flap for future cosmetic reconstruction, the TRAM flap, which, to our knowledge, is first reported herein.
